# On the Prevention of Pitting in Small-Pox

**Published:** 1863-09

**Authors:** 


### ON THE PREVENTION OF PITTING IN SMALL-POX.

While vaccination is generally regarded as the grand preventive of the disease, and all but universally practiced, it has long been felt that medical men would confer a great boon on society if they could discover some means by which the disfigurement of the face could be prevented. We believe that, by a very simple application, this desirable end has been attained in the clinical wards in the Royal Infirmary; and it is in the hope that when known it may be generally practiced that we at present draw attention to it. The application consists of a solution of india-rubber in chloroform, which is painted over the face (and neck in women,) when the eruption has become fully developed. When the chloroform has evaporated, which it very readily does, there is left a thin elastic film of india-rubber over the face. This the patient feels to be rather comfortable than otherwise, inasmuch as the disagreeable itchiness, so generally complained of, is almost entirely removed, and, what is more important, "pitting," once so common, and even now far from rare, is thoroughly prevented whenever the solution has been applied. It may be as well to state that india-rubber is far from being very soluable in chloroform, so that, in making the solution, the india-rubber must be cut into small pieces, and chloroform added till it is dissolved. The medical gentleman who has introduced this treatment has tried several other substances, but found none so generally useful. For instance, gutta percha was tried. It has the advantage of being very soluable in chloroform, and would have been a very admirable application but for the tendency it has to tear into ribands whenever the mouth is used, or even the features play. India-rubber, on the other hand, is pliable and elastic, allowing free use of the mouth without any danger (as a rule,) of its tearing off. If, however, from some cause or other, a portion is torn off, a fresh application of the solution by means of a large hair pencil remedies the defect, and the mask is once more complete. Several patients who have had this india-rubber mask applied concur in stating that they found it agreeable to wear, and their faces were perfectly free from "pitting," although some other parts of the body, such as the arms, were covered. The credit of this valuable invention and application belongs to Dr. Smart, house physician, clinical wards, Royal Infirmary; and, while he no doubt in the proper quarter will make good his claim to the honor, he will feel amply repaid by its general adoption by his medical brethern, and the consciousness that he has done something to increase the resources of the medical art.—*Scotsman.*

Dr. George writes to the *Morning Post:*—In the year 1833, I published an essay on that disease, recommending the use of prepared calamine, upon the same principle on which Dr. Smart recommends the application of a solution of india-rubber; that is, exclusion of atmospheric air. (Griffith and Ferrarand, St. Paul's Churchyard.) In a very severe case, which occurred in my practice since the publication of my essay, in which the face and throat were frightfully swollen, I dressed one-half of it with calamine powder, and the other half I pencilled over, using a flat hair pencil, with sweet oil and the white of an egg, in equal parts well mixed, three or four times a-day. No solution of india-rubber, or any other substance, would have answered the purpose better; and its application was certainly attended with more comfort than that of the use of the powder; but it is not only the pitting which is prevented by the calamine, but the rescuing the patient from a state of suffering bordering upon misery; for on the tenth day, or thereabouts, the patient has not a sore place on any part of his body, the time when, according to the usual management, his real sufferings begin. Will you permit me to offer a few practical remarks on the treatment of the disease, which might be useful to the public, and which even a nurse might act upon? Firstly.—From the commencement of the disease I would cover the whole body, face and all, lightly with the calamine, shaken through a common pepper-box; or, if hair powder be employed, the powder-puff had better be used, taking care that these powders do not remain in masses. The inflammation on each pustle is, by these applications, much lessened, a point of great consequence. Secondly.—Sprinkle about one ounce of powdered camphor every, or every two or three nights between the under sheet and blanket, the whole length of the body, putting most about the shoulders and neck. The relief obtained by this, few would credit until they had had experience. Thirdly.—In the advanced stage of the disease, should hardened incrustations have formed, they may be removed, and without much pain, too; for in one case I removed every portion of the cuticle from the whole face, forehead, and even eyelids, applied the calamine, and in a few days the cuticle was reformed without a blemish. —*Braithwaite's Retrospect.*

